# The Prognostic Nutritional Index is associated with mortality of COVID‐19 patients in Wuhan, China

**DOI:** 10.1002/jcla.23566

**Published:** 2020-09-11

**Authors:** Ruoran Wang, Min He, Wanhong Yin, Xuelian Liao, Bo Wang, Xiaodong Jin, Yao Ma, Jirong Yue, Lang Bai, Dan Liu, Ting Zhu, Zhixin Huang, Yan Kang

**Affiliations:** ^1^ Department of Critical Care Medicine West China Hospital Sichuan University Chengdu China; ^2^ COVID19 Medical Team (Hubei) of West China Hospital Sichuan University Chengdu China; ^3^ Department of Geriatrics and National Clinical Research Center for Geriatrics West China Hospital Sichuan University Chengdu China; ^4^ Center of Infectious Disease West China Hospital Sichuan University Chengdu China; ^5^ Department of Respiratory and Critical Care Medicine West China Hospital Sichuan University Chengdu China; ^6^ Department of Otolaryngology‐Head and Neck Surgery Renmin Hospital of Wuhan University Wuhan China; ^7^ Department of Obstetrics and Gynecology Renmin Hospital of Wuhan University Wuhan China

**Keywords:** COVID‐19, marker, prognosis, Prognostic nutritional index

## Abstract

**Background:**

Declared as pandemic by WHO, the coronavirus disease 2019 (COVID‐19) pneumonia has brought great damage to human health. The uncontrollable spread and poor progression of COVID‐19 have attracted much attention from all over the world. We designed this study to develop a prognostic nomogram incorporating Prognostic nutritional index (PNI) in COVID‐19 patients.

**Methods:**

Patients confirmed with COVID‐19 and treated in Renmin Hospital of Wuhan University from January to February 2020 were included in this study. We used logistic regression analysis to find risk factors of mortality in these patients. A prognostic nomogram was constructed and receiver operating characteristics (ROC) curve was drawn to evaluate the predictive value of PNI and this prognostic model.

**Results:**

Comparison of baseline characteristics showed non‐survivors had higher age (*P* < .001), male ratio (*P* = .038), neutrophil‐to‐lymphocyte ratio (NLR) (*P* < .001), platelet‐to‐lymphocyte ratio (PLR) (*P* < .001), and PNI (*P* < .001) than survivors. In the multivariate logistic regression analysis, independent risk factors of mortality in COVID‐19 patients included white blood cell (WBC) (OR 1.285, *P* = .039), PNI (OR 0.790, *P* = .029), LDH (OR 1.011, *P* < .015). These three factors were combined to build the prognostic model. Area under the ROC curve (AUC) of only PNI and the prognostic model was 0.849 (95%Cl 0.811‐0.888) and 0.950 (95%Cl 0.922‐0.978), respectively. And calibration plot showed good stability of the prognostic model.

**Conclusion:**

This research indicates PNI is independently associated with the mortality of COVID‐19 patients. Prognostic model incorporating PNI is beneficial for clinicians to evaluate progression and strengthen monitoring for COVID‐19 patients.

## Graphical abstract

AUC value of PNI for predicting morality of COVD‐19 patients is 0.849 (0.811‐0.888). While AUC value of the prognostic model composed of WBC, PNI and LDH for predicting mortality is 0.950 (0.922‐0.978). Prognostic nomogram for predicting mortality of COVID‐19 patients.Calibration plot of the prognostic model for predicting mortality of COVID‐19 patients.

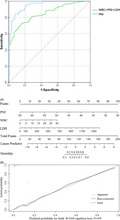

## 1 INTRODUCTION

The Corona Virus Disease 2019 (COVID‐19), initially found in Wuhan, China, spread rapidly around the world and becomes a serious global public health issue. Mainly manifested as fever, cough, and fatigue, nearly half of COVID‐19 patients would develop dyspnea with concurrent hypoxia one week after onset.^1^, ^2^, ^3^ In addition to impaired respiratory function, function of other organs could also be damaged. Complications including cardiac injury, acute kidney injury, acute gastrointestinal injury, coagulopathy, and liver dysfunction are relatively common in critically ill cases^4^, ^5^ and were confirmed associated with poor outcome in COVID‐19 patients.^6^, ^7^, ^8^, ^9^, ^10^ These organs damage is considered resulting from the cytokine release syndrome (CRS) which plays pivotal role in the progression of COVID‐19 patients.^11^, ^12^ One of the most core cytokines in CRS is the Interkulin‐6 (IL‐6), which has been acknowledged playing an important role in acute inflammation of various diseases.^13^, ^14^, ^15^ The release of excessive cytokines including IL‐6 in COVID‐19 patients is attributable to the activation of innate and adaptive immune system caused by SARS‐CoV‐2.^16^

The dysregulation of immune response and excessive inflammation actually is key element of pathogenesis in COVID‐19.^17^, ^18^ And many immunity and inflammation‐related markers including C‐reactive protein (CRP), IL‐6, neutrophil‐to‐lymphocyte ratio (NLR), and platelet‐to‐lymphocyte ratio have been confirmed associated with disease severity and mortality of COVID‐19 patients.^19^, ^20^, ^21^, ^22^, ^23^

The Prognostic Nutritional Index (PNI), a common marker of immune and inflammatory status, has been proved of prognostic value in various clinical settings including cardiovascular diseases, infectious diseases, and cancer.^24^, ^25^, ^26^, ^27^, ^28^, ^29^ Incorporating effects of both lymphocyte and albumin, low PNI could indicate poor prognosis of patients. We designed this study to explore the prognostic value of PNI in COVID‐19 patients.

## 2 MATERIALS AND METHODS

### 2.1 Subjects

Patients admitted to Renmin Hospital of Wuhan University for COVID‐19 from January 30 to February 24, 2020 were eligible in this study. The diagnose of COVID‐19 patients was confirmed by the positive result for SARS‐Cov‐2 RNA in nasopharyngeal swabs by using real‐time fluorescence reverse transcription‐polymerase chain reaction (RT‐PCR). Patients died on admission and transferred from other hospitals were excluded from this study. Finally, a total of 450 patients were included in this single‐center study.

### 2.2 Data collection

Demographical and clinical data of included patients were collected by searching records in electronic medical record system (EMRS). Complicated underlying diseases in admission including hypertension, cardiovascular disease, chronic respiratory or liver disease, and cancer were included as potential risk factors in this study. Results of laboratory tests were obtained by analyzing the blood sample collected on admission. The PNI was calculated as serum albumin (g/L) + 5 × lymphocyte count (10^9^/L). In addition, neutrophil‐to‐lymphocyte ratio (NLR) and platelet‐to‐lymphocyte ratio (PLR) were also calculated and included as potential risk factors. The primary outcome of this study was in‐hospital mortality acquired by following up from admission to discharge. This study was approved by the ethics committee of West China hospital of Sichuan University and Renmin Hospital of Wuhan University. The whole process of this study was accorded with the Declaration of Helsinki. Patients included in this observational study have signed an informed consent.

### 2.3 Statistical analysis

**FIGURE 1:**
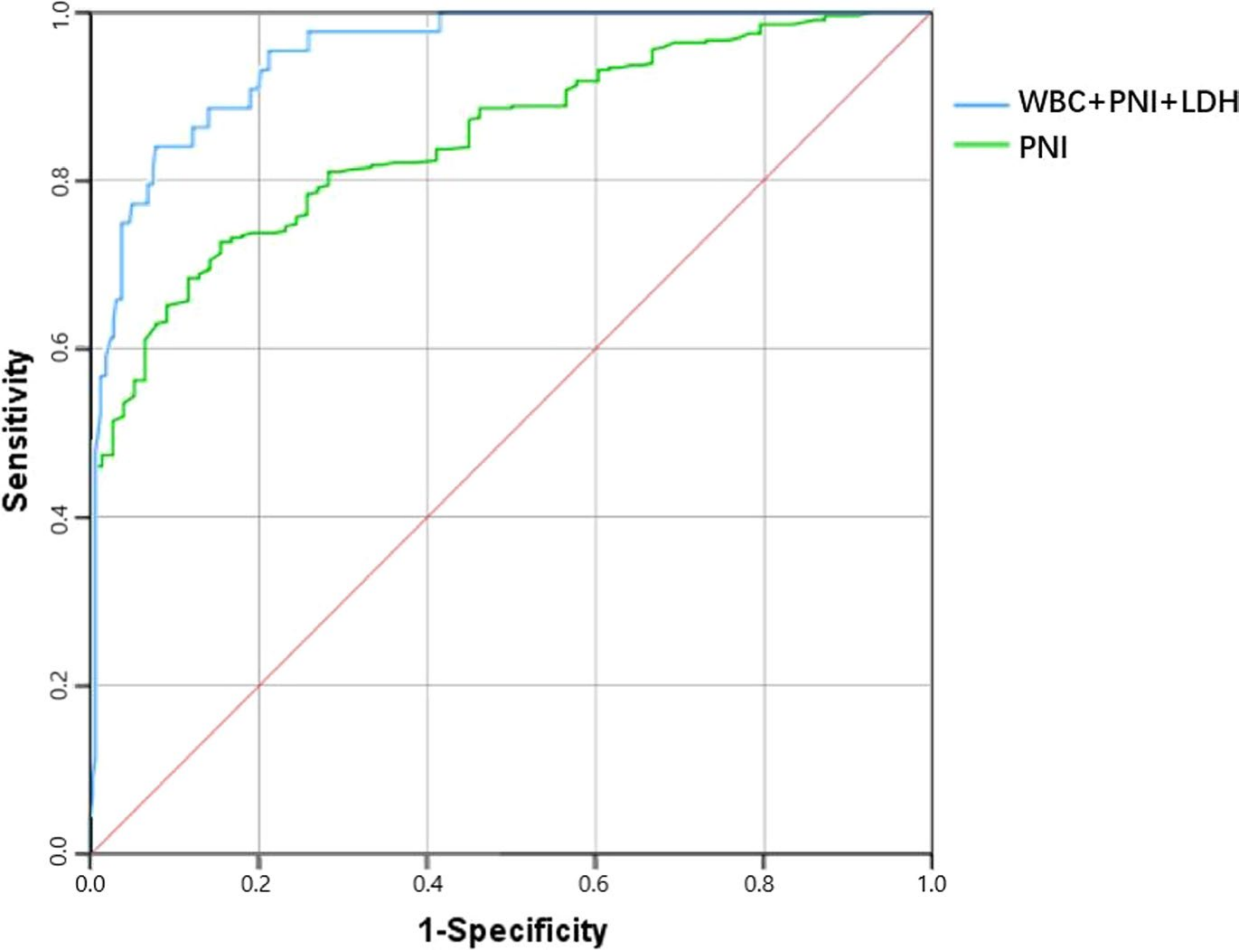
Receiver operating characteristics curve of Prognostic nutritional index and prognostic model for predicting mortality in COVID‐19 patients

We used Kolmogorov‐Smirnov test to verify the normality of variables. Normally distributed variables were shown as mean ± standard deviation while non‐normally distributed variables were shown as median (interquartile range). And categorical variables were presented as the form of numbers (percentage). We respectively performed independent Student's *t* test and Mann‐Whitney *U* test to analyze differences between two groups of normally distributed and non‐normally distributed variables. Chi‐square test was performed to examine the difference of categorical variables. Then, univariate and multivariate logistic regression were sequentially performed to explore risk factors of mortality in COVID‐19 patients. By multivariate logistic regression, we developed a prognostic nomogram using the rms package in R project. The receiver operating characteristic (ROC) curves were drawn, and area under the ROC curves (AUC) were calculated to evaluate the discrimination ability of PNI and the prognostic nomogram. Finally, we evaluate the stability of the prognostic nomogram by internal validation with 1000 bootstrap samples. Calibration plots were drawn to analyze the consistency between observed probability and predicted probability of poor outcome in COVID‐19 patients (Figure 1).

A *P* value <.05 was considered to be statistically significant. SPSS 22.0 Windows software (SPSS, Inc) and R (version 3.6.1; R Foundation) were used for all statistical analysis and figure drawing.

## 3 RESULTS

### 3.1 Baseline characteristics between survivors and non‐survivors in COVID‐19 patients

A total of 450 patients confirmed with COVID‐19 were included in this study. There were 372 survivors and 78 non‐survivors with a mortality rate of 17.3% (Table 1.). Compared with survivors, non‐survivors had significant higher age (71 vs 55, *P* < .001) and male ratio (56.4% vs 43.5%, *P* = .038). In terms of underlying diseases, hypertension (41.0% vs 15.3%, *P* < .001), cardiovascular disease (10.3% vs 1.9%, *P* = .001), and chronic respiratory disease (16.7% vs 4.6%, *P* = .001) were more frequently observed in non‐survivors group. Records of vital signs in admission showed that non‐survivors had faster heart rate (88 vs 83, *P* = .006) and respiratory rate (22 vs 20, *P* < .001). Observing outcomes of blood biochemistry and routine, we found that non‐survivors had higher level of white blood cell (WBC), neutrophil, alanine aminotransferase (ALT), aspartate aminotransferase (AST), alkaline phosphatase (ALP), lactate dehydrogenase (LDH), blood urea nitrogen (BUN), serum creatinine, C‐reactive protein (CRP) (Table 1.). Whereas the level of lymphocyte, platelet, albumin was significantly lower in non‐survivors. In addition, the value of PNI (36.8 vs 44.3, *P* < .001) was lower in non‐survivors while value of NLR (12.41 vs 2.82, *P* < .001) and PLR (237.32 vs 173.29, *P* < .001) were higher in survivors. Coagulation test indicated that non‐survivors had higher level of international normalized ratio (INR), prothrombin time (PT), thrombin time (APTT), and D‐dimer than survivors. Compared with survivors, non‐survivors had shorter length of hospital stay (LOS) (6 vs 9, *P* < .001).

**TABLE 1:** Demographical and clinical characteristics between survivors and non‐survivors

	All patients (N = 450)	Survivors (N = 372, 78%)	Non‐survivors (N = 78, 17.3%)	*P* value
Age	58 (41‐70)	55 (38‐67)	71 (63‐78)	**<.001**
Sex (male)	206 (45.8%)	162 (43.5%)	44 (56.4%)	**.038**
Comorbidity				
Diabetes mellitus	26 (5.8%)	19 (5.1%)	7 (9.0%)	.185
Hypertension	89 (19,8%)	57 (15.3%)	32 (41.0%)	**<.001**
Cardiovascular disease	15 (3.33%)	7 (1.9%)	8 (10.3%)	**.001**
Chronic respiratory disease	30 (6.7%)	17 (4.6%)	13 (16.7%)	**.001**
Chronic liver disease	20 (4.4%)	14 (3.8%)	6 (7.7%)	.133
Cancer	8 (1.8%)	5 (1.3%)	3 (3.8%)	.146
Vital signs in admission				
Body temperature (°C)	36.7 (36.5‐37)	36.7 (36.5‐36.7)	36.7 (36.5‐37.0)	.697
MAP (mmHg)	93.3 (86.3‐97.7)	93.3 (86.7‐97.2)	93.2 (84.3‐102.2)	.576
Heart rate (bps)	84 (78‐93)	83 (77‐83)	88 (79‐104)	**.006**
Respiratory rate (min^−1^)	20 (18‐21)	20 (18‐20)	22 (18‐28)	**<.001**
Laboratory tests				
WBC (×10^9^/L)	5.76 (4.29‐7.91)	5.43 (4.11‐7.20)	9.04 (6.35‐12.47)	**<.001**
Neutrophil (×10^9^/L)	3.89 (2.61‐6.33)	3.39 (2.48‐5.02)	7.41 (4.96‐11.32)	**<.001**
Lymphocyte (×10^9^/L)	1.13 (0.77‐1.55)	1.21 (0.90‐1.62)	0.66 (0.40‐0.92)	**<.001**
Monocyte (×10^9^/L)	0.43 (0.30‐0.59)	0.45 (0.32‐0.59)	0.38 (0.26‐0.62)	.078
Platelet (×10^9^/L)	212 (157‐272)	220 (165‐283)	172 (118‐221)	**<.001**
Hemoglobin (g/L)	127 (115‐138)	126 (115‐137)	131 (117‐140)	**.150**
Albumin (g/L)	37.1 (33.4‐40.2)	38.0 (34.6‐41.0)	33.2 (31.2‐35.4)	**<.001**
Globulin (g/L)	24.1 (21.7‐27.8)	24.0 (21.7‐27.6)	24.9 (21.8‐29.0)	.248
PNI	42.96 ± 6.25	44.25 ± 5.85	36.80 ± 4.06	**<.001**
NLR	3.21 (1.94‐7.11)	2.82 (1.69‐5.01)	12.41 (7.16‐18.75)	**<.001**
PLR	182.42 (138.05‐274.66)	173.29 (132.35‐252.22)	237.32 (160.15‐400.96)	**<.001**
ALT (U/L)	25 (16‐39)	24 (16‐39)	27 (20‐46)	**.05**
AST (U/L)	26 (20‐40)	24 (19‐36)	43 (30‐63)	**<.001**
ALP (U/L)	61 (50‐80)	60 (49‐74)	74 (53‐102)	**.001**
LDH (U/L)	246 (190‐311)	229 (186‐288)	544 (372‐718)	**<.001**
BUN (mmol/L)	4.70 (3.70‐6.60)	4.37 (3.61‐5.75)	7.80 (5.10‐11.95)	**<.001**
Serum creatinine (umol/L)	59 50‐73)	58 (49‐70)	67.5 (52‐86)	**.001**
CRP (mg/L)	22.1 (5‐65.4)	11.2 (5.0‐47.9)	92.2 (53.8‐167.6)	**<.001**
PCT (ng/L)	1.11 (0.12‐4.4)	2.50 (0.11‐4.40)	0.42 (0.16‐3.20)	.733
INR	1.03 (0.97‐1.09)	1.03 (0.96‐1.08)	1.09 (1.03‐1.19)	**<.001**
PT (s)	12 (11.4‐12.7)	11.9 (11.3‐12.5)	12.0 (12.7‐13.8)	**<.001**
TT (s)	17.8 (17‐18.7)	17.7 (17.0‐18.6)	17.9 (16.8‐19.6)	.135
APTT (s)	28.1 (25.9‐30.5)	27.7 (25.7‐29.9)	29.7 (27.3‐32.3)	**<.001**
FIB (g/L)	4.1 (3.04‐5.12)	4.09 (3.09‐4.97)	4.44 (2.75‐5.70)	.228
D‐dimer (mg/L)	0.79 (0.38‐2.45)	0.63 (0.33‐1.77)	4.98 (0.96‐17.57)	**<.001**
Length of hospital stay	9 (5‐13)	9 (5‐14)	6 (4‐10)	<.001

Abbreviations: ALP, alkaline phosphatase; ALT, alanine aminotransferase; APTT, activated partial thromboplastin time; AST, aspartate aminotransferase; BUN, blood urea nitrogen; CRP, C‐reactive protein; FIB, fibrinogen; INR, international normalized ratio; LDH, lactate dehydrogenase; MAP, mean arterial pressure; NLR, neutrophil‐to‐lymphocyte ratio; PCT, procalcitonin; PLR, platelet‐to‐lymphocyte ratio; PNI, prognostic nutritional index; PT, prothrombin time; TT, thrombin time; WBC, white blood cell.

### 3.2 Univariate and multivariate logistic regression analysis of risk factors for mortality in COVID‐19 patients

The statistically different characteristics in baseline comparison were included into logistic regression analysis. Results of univariate logistic regression analysis showed that most of variables significant in baseline comparison were still statistically significant in univariate logistic regression analysis except for ALT (OR 1.006, *P* = .094) and INR (OR 1.285, *P* = .282) (Table 2.). Then, multivariate logistic regression analysis indicated only WBC (OR 1.285, *P* = .039), PNI (OR 0.790, *P* = .029), and LDH (OR 1.011, *P* < .015) were independent risk factors of mortality in COVID‐19 patients.

**TABLE 2:** Univariate and multivariate logistic regression analysis of risk factors for in‐hospital mortality in COVID‐19 patients

	Univariate analysis			Multivariate analysis		
	OR	95 Cl%	*P*	OR	95 Cl%	*P*
Age	1.072	1.051‐1.094	<.001	0.984	0.929‐1.042	.574
Sex (male)	1.678	1.025‐2.744	.039	0.881	0.207‐3.741	.863
Hypertension	3.844	2.258‐6.545	<.001	2.304	0.487‐10.900	.292
Cardiovascular disease	5.959	2.093‐16.963	.001	2.132	0.022‐208.421	.746
Chronic respiratory disease	4.176	1.936‐9.011	<.001	4.173	0.559‐31.159	.164
Heart rate (bps)	1.024	1.008‐1.041	.003	1.020	0.979‐1.063	.340
Respiratory rate	1.124	1.076‐1.173	<.001	1.101	0.955‐1.269	.184
WBC	1.293	1.198‐1.395	<.001	1.285	1.012‐1.632	**.039**
PNI	0.755	0.706‐0.807	<.001	0.790	0.639‐0.976	**.029**
NLR	1.210	1.155‐1.266	<.001	0.868	0.711‐1.059	.164
PLR	1.004	1.002‐1.005	<.001	1.003	0.999‐1.007	.154
ALT	1.006	0.999‐1.013	.094	1.009	0.987‐1.031	.427
AST	1.021	1.012‐1.030	<.001	0.988	0.948‐1.030	.566
ALP	1.012	1.006‐1.018	<.001	1.008	0.991‐1.026	.354
LDH	1.009	1.006‐1.011	<.001	1.011	1.002‐1.019	**.015**
BUN	1.144	1.086‐1.205	<.001	1.235	0.970‐1.573	.087
Serum creatinine	1.007	1.003‐1.012	.002	1.006	0.941‐1.075	.864
CRP	1.020	1.015‐1.025	<.001	1.013	0.999‐1.026	.063
INR	1.285	0.814‐2.029	.282	0.634	0.006‐61.805	.845
PT	2.318	1.761‐3.052	<.001	1.155	0.447‐2.988	.766
APTT	1.154	1.069‐1.245	<.001	1.057	0.872‐1.280	.574
D‐dimer	1.048	1.024‐1.073	<.001	0.988	0.941‐1.038	.639

Abbreviations: ALP, alkaline phosphatase; ALT, alanine aminotransferase; APTT, activated partial thromboplastin time; AST, aspartate aminotransferase; BUN, blood urea nitrogen; CRP, C‐reactive protein; FIB, fibrinogen; INR, international normalized ratio; LDH, lactate dehydrogenase; NLR, neutrophil to lymphocyte ratio; PLR, platelet to lymphocyte ratio; PNI, prognostic nutritional index; PT, prothrombin time; WBC, white blood cell.

### 3.3 Predictive value of PNI and prognostic model

**FIGURE 2:**
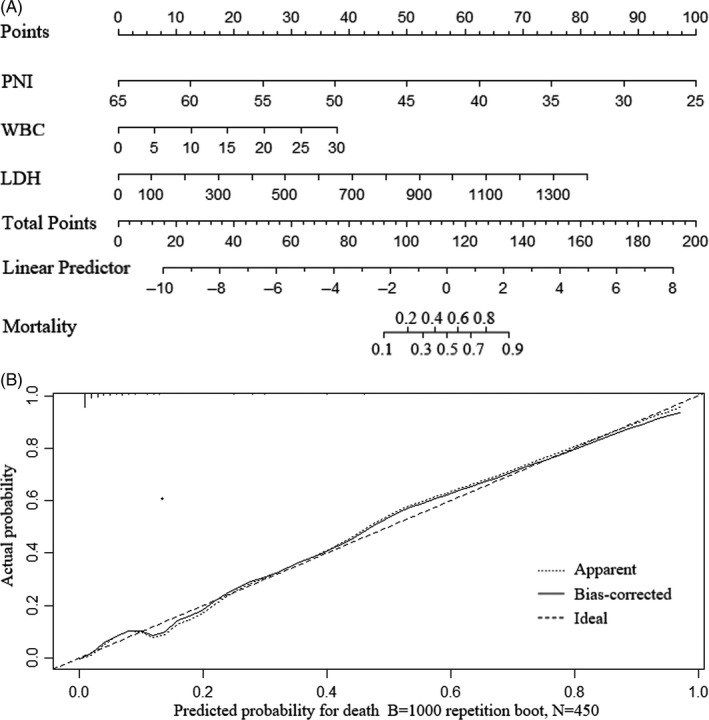
A, Nomogram of the prognostic model for predicting mortality in the study cohort. B, Calibration plot for predicting mortality in the study cohort

We constructed prognostic model utilizing independent risk factors in multivariate logistic regression analysis. Consisted of WBC, PNI, LDH, the constructed prognostic model had an AUC value of 0.950 (95% Cl 0.922‐0.978, *P* < .001) (Table 3) (Figure 1). The AUC value of PNI for predicting mortality was 0.849 (95% Cl 0.811‐0.888, *P* < .001). Compared to single PNI value, the prognostic model may perform better in predicting outcome of COVID‐19 patients (*Z* = 4.137, *P* < .05). For visualization and convenient clinical use of the prognostic model, nomogram was built incorporating these three factors (Figure 2A). The stability of this model was internally validated with 1000 bootstrap samples. The calibration plot showed a good consistency between the prediction by nomogram and actual observation (Figure 2B).

**TABLE 3:** Predictive value of PNI and the Prognostic model

	AUC	95% Cl	Sensitivity	Specificity
PNI	0.849	0.811‐0.888	0.726	0.846
Prognostic model	0.950	0.922‐0.978	0.841	0.922

Note

The prognostic model is consisted of WBC, PNI, LDH.

Abbreviations: AUC, area under the receiver operating characteristics curve; Cl, confidence interval.

## 4 DISCUSSION

The mortality rate of previous studies reported ranged from 1% to 28.3%.^1^, ^5^, ^30^, ^31^ In this study, there were 78 patients suffered poor outcome with mortality rate of 17.3%. This difference might be attributable to the heterogeneity of included patients, differences in medical treatment level and medical resources. Our results showed non‐survivors had older age, higher male ratio, and higher incidence of comorbidities. And underlying diseases including hypertension, cardiovascular disease, and chronic respiratory disease were found associated with mortality in univariate logistic regression analysis. These findings were consistent with results of previous studies.^32^, ^33^, ^34^ Moreover, higher NLR and PLR were found positively associated with mortality in univariate analysis. However, after adjustments, only WBC, PNI, and LDH were independently correlated with outcome of COVID‐19 patients in multivariate logistic regression analysis.

The PNI, calculated from albumin and lymphocyte levels, is an objective reflection of inflammatory and nutritional status. And it has been confirmed being of prognostic value in various settings such as cardiovascular disease and cancer.^24^, ^27^, ^35^ In our study, the level of albumin was significantly lower in non‐survivors compared with survivors. And previous studies have shown that albumin level was inversely associated with unfavorable progression and outcome in COVID‐19 patients.^36^, ^37^ Low level of albumin in non‐survivors might be attributable to intubation induced inadequate intake, reduced synthesis caused by liver dysfunction and increased consumption due to organ damage. The correlation between poor outcome and low albumin level could be mediated by several mechanisms. Firstly, synthesized by hepatocytes, albumin level is an indicator of liver function. Inflammatory cytokines such as interkulin‐6 (IL‐6) and tumor necrosis factor‐α (TNF‐α) could inhibit synthesis ability of hepatocytes so that the serum level of albumin decreases. ^38^ It is the cytokine storm, which is characterized as a large release of cytokines including interkulin‐1 (IL‐1), IL‐6, TNF‐α, monocyte chemotactic protein 1 (MCP‐1), inducible protein‐10 (IP‐10), Interferon‐γ (IFN‐γ), and granulocyte colony‐stimulating factor (G‐CSF), is responsible for the severe organ damage in COVID‐19 patients.^2^, ^39^ It has been confirmed that cytokines such as IL‐1ra, IL‐2R, IL‐6, IL‐10, TNF‐α, IP‐10, and MCP‐3 were associated with severity and progression in COVID‐19 patients.^40^, ^41^ Therefore, the decreased level of albumin might indicate severe degree of cytokine storm and organ damage including liver dysfunction in COVID‐19 patients. Secondly, low level of albumin could lead to the exudation of intravascular fluid which exacerbate the severity of pulmonary edema. The serum albumin level has been verified inversely associated with development of acute respiratory distress syndrome (ARDS) in COVID‐19 patients.^42^ The development of ARDS is undoubtedly a risk factor of poor outcome in COVID‐19 patients.^4^, ^5^, ^42^ Consequently, low albumin level is not conducive to favorable outcome by impairing pulmonary function in COVID‐19 patients. Finally, as a common marker of nutritional status, low albumin level could indicate heavy consumption status caused by tissue damage and hypermetabolism in critical patients. Reflected by albumin level, the poor nutritional status in turn is not conducive to tissue repair and recovery of COVID‐19 patients. As another important component of PNI, the count of lymphocyte was significantly lower in non‐survivors. The decrease of T cells especially CD3^+^, CD4^+^, and CD8^+^T cells accounts for a majority of reduced peripheral lymphocytes in COVID‐19 patients.^43^ Decreased CD4^+^ and CD8^+^ T cells along with excessive activation of themselves is a key characteristics of immunocompromise and correlated with adverse progression in COVID‐19 patients.^44^ It is speculated that direct attack from virus to lymphocyte, antigen presenting cells (APC) dysfunction and apoptosis due to excessive release of cytokines could result in the decrease of T cells.^45^, ^46^, ^47^ Whatever, the lymphopenia has been confirmed as an independent risk factor of mortality in COVID‐19 patients.^47^ And other inflammatory markers incorporating lymphocyte such as NLR and PLR are also associated with severity and outcome in COVID‐19 patients.^19^, ^20^, ^48^ The decreased lymphocyte might be considered as a reflection of impaired immune function and sharply increasing cytokines. The PNI, composed of albumin level and lymphocyte count, could reflect nutritional and inflammatory status more comprehensively in COVID‐19 patients.

The WBC count and LDH were another two significant factors in multivariate logistic regression analysis. Both of them were significantly higher in non‐survivors than survivors.

The increase of WBC was mainly attributable to the obviously increasing neutrophils. A study has demonstrated that neutrophilia was valuable in predicting unfavorable clinical outcomes in COVID‐19 patients.^8^ Actually, neutrophils are the major source of chemokines and cytokines in the course of some diseases such as sepsis.^49^ Previous researches about Middle East respiratory syndrome showed that extension and degree of pulmonary neutrophils infiltration and number of peripheral neutrophils were associated with the severity of lung damage.^50^, ^51^ Therefore, a reasonable inference is that increasing WBC count especially the neutrophil is positively correlated with severe pulmonary lesion which could aggravate the adverse progress in COVID‐19 patients. The LDH, usually acknowledged as a marker of tissue injury, has been proved of prognostic value in COVID‐19 patients.^43^ And serum LDH level is significantly correlated with indicators of inflammation, cardiac and liver injury such as AST, CRP, and brain natriuretic peptide (BNP).^43^ Therefore, the LDH level could indicate severity of systemic inflammation and organ damage associated with clinical outcome in COVID‐19 patients.

Our prognostic model composed of WBC, PNI, and LDH could objectively reflect inflammatory and immune status more comprehensively than single value of these factors in COVID‐19 patients. This model had good performance in predicting mortality of COVID‐19 patients with high distinguishing ability and stability. In addition, compared with other expensive and time‐consuming prognostic biomarkers, those three components of this model were readily obtained from results of daily blood routine and blood biochemistry without too much cost.

This study had several limitations. Firstly, the selection bias could not be avoided due to the nature of single institutional study. And the number of included patients was relatively small. Therefore, the effectiveness of our prognostic model should be further testified in other medical centers with larger sample size. Secondly, significant cytokines correlated with severity and outcome were not evaluated and recorded. The real effect of risk factors in this study may be confounded by these cytokines.

## 5 CONCLUSIONS

The PNI is inversely associated with outcome in COVID‐19 patients. Prognostic model incorporating PNI shows good performance in predicting outcome of COVID‐19 patients. The nomogram of our model provides physicians with visual prognostic assessment for COVID‐19 patients.

## CONFLICT OF INTEREST

The authors have no conflicts of interest to disclose.

## AUTHORS CONTRIBUTIONS

Ruoran Wang and Min He conceived and designed the study. Dan Liu, Ting Zhu, Yao Ma, and Lang Bai involved in data acquisition. Xuelian Liao and Bo Wang analyzed and interpreted the data. Xiaodong Jin and Wanhong Yin involved in statistical analysis. Ruoran Wang and Zhixin Huang drafted the manuscript. Yan Kang and Jirong Yue revised the manuscript for important intellectual content. All authors read and approved the final manuscript.

## ETHICAL APPROVAL

This study was approved by the ethics committee of West China hospital of Sichuan University and Renmin Hospital of Wuhan University.
